# In-hospital complications of hybrid vs endocardial atrial fibrillation ablation

**DOI:** 10.1016/j.hroo.2025.05.023

**Published:** 2025-05-23

**Authors:** Mahmoud Eisa, Nauman Naeem, Hossam Elbenawi, Asmaa Ahmed, Andrew Takla, Amir Hanafi, Abhishek J. Deshmukh, Christopher.V. DeSimone, Mohan Rao

**Affiliations:** 1 Department of Internal Medicine, Rochester General Hospital, Rochester, New York; 2 Department of Cardiovascular Diseases, Mayo Clinic, Rochester, Minnesota; 3 Department of Internal Medicine, Unity Hospital, Rochester, New York; 4 Sands Constellation Heart Institute, Rochester Regional Health, Rochester, New York

**Keywords:** Atrial fibrillation, Hybrid ablation, Endocardial ablation, MACE, Propensity score weighting, NIS database

## Abstract

**Background:**

Hybrid atrial fibrillation (AF) ablation has been used to achieve rhythm control in AF management. It requires both epicardial and endocardial ablation. There is limited evidence regarding the safety of this procedure.

**Objective:**

The study aimed to compare in-hospital complication rates between hybrid AF ablation and endocardial catheter ablation (ECA) using a nationally representative dataset.

**Methods:**

Using the National Inpatient Sample, we identified patients who underwent AF ablation between 2017 and 2022. The cohort was divided into 2 groups; those who underwent ECA and those who underwent hybrid AF ablation. The primary outcome was major adverse cardiovascular events (MACEs).

**Results:**

After propensity score weighting, hybrid ablation was associated with a significantly lower MACE rate (3.1% vs 5.6%, *P =* .0036). Rates of cardiac complications (8.8% vs 7.5%, *P =* .594), infections (2.7% vs 3.7%, *P =* .595), and length of stay (2.96 ± 0.30 vs 3.19 ± 2.45 days, *P =* .285) were comparable between groups. Hybrid ablation was associated with higher rates of hemorrhagic complications (12.7% vs 4.0%, *P <* .001), with a comparable rate of blood transfusion, whereas pulmonary complications showed a trend toward an increase (3.8% vs 1.5%, *P =* .099).

**Conclusion:**

Hybrid AF ablation was associated with a significantly lower MACE rate compared with ECA, contrasting with earlier studies that suggested higher risk. This shift likely reflects improvements in technique and more refined patient selection. While rates of pulmonary and hemorrhagic complications were higher with hybrid ablation, transfusion needs remained similar between groups.

**Key Findings**

▪ Hybrid ablation was associated with a significantly lower risk of in-hospital major adverse cardiovascular events (MACEs) compared with endocardial catheter ablation, based on propensity score-weighted analysis of a large national dataset.
▪ Rates of cardiac, infectious, and thromboembolic complications were comparable between the 2 groups.
▪ Hybrid ablation was associated with increased rates of hemorrhagic and pulmonary complications, though blood transfusion requirements were similar.
▪ Multivariable analysis confirmed the robustness of these findings, reinforcing the importance of patient selection and identifying coagulopathy and fluid or electrolyte disorders as independent predictors of MACE.
▪ These results highlight the evolving safety profile of hybrid ablation in contemporary practice and support its use in appropriately selected patients.

## Introduction

AF ablation has been widely used to achieve rhythm control in atrial fibrillation (AF) patients because of its relative safety and efficacy.^1^^,^^2^ AF ablation can be done either via percutaneous, catheter-based endocardial catheter ablation (ECA), or surgically, such as with the Cox-Maze IV procedure.^3^ The Cox-Maze IV procedure achieves favorable long-term outcomes, with nearly 77% of patients remaining free from AF at 10 years.^4^ Surgical AF ablation is typically performed on patients with concomitant cardiac disease undergoing cardiac surgery.^5^ Total thoracoscopic maze is a minimally invasive technique of epicardial ablation through a closed chest approach. Although its major limitation is the inability to create transmural lesions necessary for electrical isolation, it has expanded the options for AF ablation by allowing direct surgical visualization minimizing the risk of esophageal injury, without the risks associated with open chest surgery.^6^ These limitations with epicardial ablation prompted the development of a hybrid approach, which combines surgical epicardial ablation (via thoracoscopy or a laparoscopic subxiphoid approach) with transvenous ECA. The hybrid method allows for endocardial ablation to be conducted either as a single-stage procedure or as a 2-stage process following epicardial ablation.^7^ A crucial element of this approach is its integration of both epicardial and endocardial ablation, which enables effective targeting of key AF drivers beyond the pulmonary veins, such as the left atrial posterior wall, left atrial appendage, and ganglionated plexi ablation, all of which are involved in non-paroxysmal AF.^7^ The goal of our study is to compare the outcomes of single-stage hybrid ablation vs conventional ECA using data from the National Inpatient Sample (NIS) database.

## Methods

### Data source

We conducted this study using the NIS data from 2017 to 2022. The NIS is a database provided by the Agency of Healthcare Research and Quality of the United States (US) through a federal–state–industry partnership. The NIS is the largest publicly available all-payer inpatient care database in the US, comprising data on over 7 million unweighted hospital stays and approximately 35 million weighted hospital encounters annually. It incorporates a stratified sample of 20% non-federal US community hospitals representing nearly 95% of the US population.^8^

### Study population

**Figure 1:**
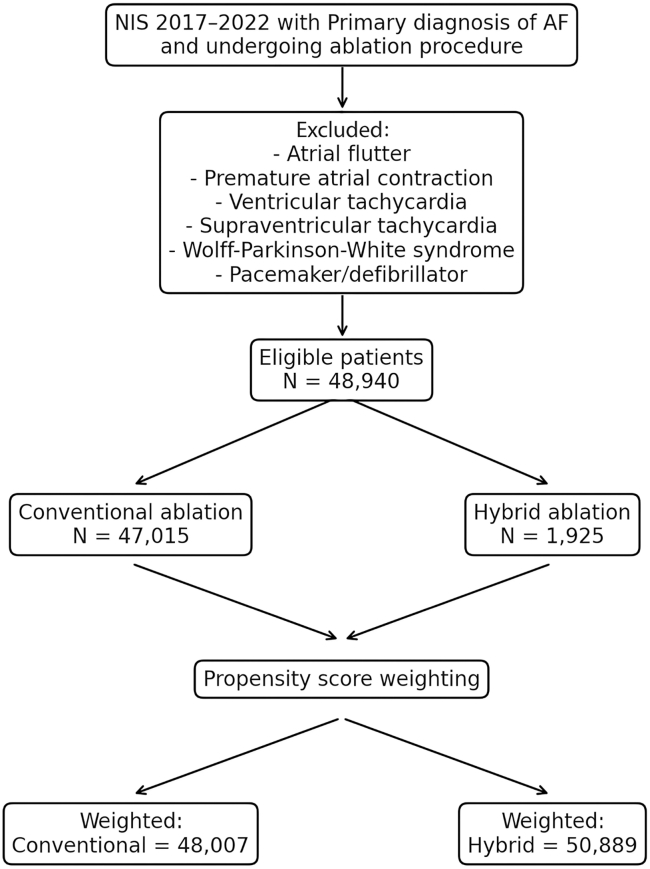
Study design. AF = atrial fibrillation; NIS = National Inpatient Sample.

Using the International Classification of Diseases, Tenth Revision (ICD-10), Clinical Modification/ICD-10, Procedure Coding System codes, we identified all patients ≥ 18 years with a primary admission diagnosis of AF who underwent an AF ablation procedure during the index hospitalization. We excluded patients with a secondary diagnosis of atrial flutter, premature atrial contraction, ventricular tachycardia, supraventricular tachycardia, non-specific tachycardia, or Wolff-Parkinson-White syndrome. We also excluded patients who underwent pacemaker or defibrillator placement during the index hospitalization. Included patients were further classified as undergoing ECA or hybrid AF ablation (Figure 1).

Hybrid AF ablation was defined as a procedure in which patients underwent both endocardial (percutaneous) ablation and epicardial (percutaneous endoscopic) ablation within the same hospitalization. The coding for hybrid AF ablation was derived from the insurance company’s coding guide and applied to all AF ablation-related codes.^9^^,^^10^ The specific ICD-10, Clinical Modification and ICD-10, Procedure Coding System codes used in this analysis are detailed in Supplemental Tables 1 and 2. We included only single-stage hybrid ablation cases performed during the same hospital stay, as the NIS only captures inpatient encounters and cannot track procedures done across multiple admissions or in outpatient settings. Because the NIS does not specify the type of hybrid ablation, we focused on single-stage inpatient cases, which likely reflect mostly convergent procedures—the most common hybrid approach in the US.

### Study variables

Baseline characteristics included demographic variables (age and sex) and baseline comorbid medical conditions such as hypertension, diabetes mellitus, hyperlipidemia, obesity, smoking, liver failure, and renal failure. Definitions of diagnoses and baseline characteristics are detailed in Table 1. We analyzed the rate of complications and the trend of procedures over the studied years.

**Table 1:** Baseline characteristics

Variables	Subcategory	Regular AF ablation (47015)	Hybrid AF ablation (1925)	*P*-value
Age (mean ± SD)		67.8 ± 7.09	65.7 ± 5.95	< .001
Women (%)		20,216 (43.0%)	558 (29%)	< .001
Congestive heart failure (%)		21,124 (44.93%)	825 (42.86%)	.570
Hypertension (%)		36,836 (78.35%)	1535 (79.74%)	.631
Diabetes mellitus (%)		12,271 (26.1%)	550 (28.57%)	.337
Peripheral vascular disease (%)		6389 (13.59%)	185 (9.61%)	.002
Hypothyroidism (%)		7969 (16.95%)	305 (15.84%)	.690
Renal failure (%)		8058 (17.14%)	275 (14.29%)	.197
Liver disease (%)		1157 (2.46%)	20 (1.04%)	.003
Rheumatic disease (%)		1302 (2.77%)	65 (3.38%)	.391
Coagulopathy (%)		2158 (4.59%)	110 (5.71%)	.360
Obesity (%)		12,971 (27.59%)	805 (41.82%)	7.33E-6
Weight loss (%)		705 (1.5%)	0 (0%)	.
Fluid and electrolyte disorders (%)		6972 (14.83%)	370 (19.22%)	.037
Alcohol abuse (%)		1016 (2.16%)	< 55 (< 3%)	.899
Valvular disease (%)		8862 (18.85%)	355 (18.44%)	.836
Depression (%)		4006 (8.52%)	135 (7.01%)	.381
Smoking (%)		3498 (7.44%)	165 (8.57%)	.326
Dementia (%)		710 (1.51%)	0 (0%)	.
COPD (%)		10,104 (21.49%)	315 (16.36%)	.004
Pulmonary circulation disorders (%)		2938 (6.25%)	75 (3.90%)	.051
Elective admission (%)		27,175 (57.8%)	1775 (92.21%)	4.72E-17
AF type∗				< .001
	Paroxysmal AF	19,605 (41.7%)	244 (12.7%)	
	Persistent AF	21,956 (46.7%)	1600 (83.1%)	
	Chronic AF	1834 (3.9%)	31 (1.6%)	
	Other AF	3620 (7.7%)	50 (2.6%)	
Race (%)				.0009
	White	39,022 (83.0%)	1694 (88%)	
	Black	2351 (5.0%)	77 (4%)	
	Hispanic	2821 (6.0%)	77 (4%)	
	Other	2821 (6.0%)	77 (4%)	
Charlson Comorbidity Index score (%)				.2678
	Score: 0	13,634 (29.0%)	616 (32%)	
	Score: 1	12,224 (26.0%)	558 (29%)	
	Score: 2	8463 (18.0%)	327 (17%)	
	Score: 3+	12,694 (27.0%)	443 (23%)	
Median household income quartile (%)				.0034
	Quartile: 1 lowest	10,813 (23.0%)	308 (16%)	
	Quartile: 2	11,284 (24.0%)	520 (27%)	
	Quartile: 3	11,754 (25.0%)	597 (31%)	
	Quartile: 4 Highest	13,164 (28.0%)	500 (26%)	
Insurance (%)				.0006
	Medicare	30,090 (64.0%)	1078 (56%)	
	Medicaid	2821 (6.0%)	77 (4%)	
	Private	13,634 (29.0%)	751 (39%)	
	Other	470 (1.0%)	<55 (<3%)	
Hospital region (%)				.1808
	Northeast	13,164 (28.0%)	424 (22%)	
	Midwest	7993 (17.0%)	270 (14%)	
	South	20,216 (43.0%)	943 (49%)	
	West	6112 (13.0%)	270 (14%)	
Hospital bed size (%)				.3321
	Small	5172 (11.0%)	173 (9%)	
	Medium	13,634 (29.0%)	500 (26%)	
	Large	28,209 (60.0%)	1251 (65%)	
Hospital location (%)				.3809
	Rural	940 (2.0%)	<55 (<3%)	
	Urban	46,075 (98.0%)	1906 (99%)	
Hospital teaching status (%)				.4863
	Non-teaching	5642 (12.0%)	212 (11%)	
	Teaching	41,373 (88.0%)	1713 (89%)	

AF = atrial fibrillation; COPD = chronic obstructive pulmonary disease; SD = standard deviation.

∗ AF type is based on admission diagnosis.

### Study outcomes

The primary outcome is the major adverse cardiac events (MACEs), defined as the composite of all-cause mortality, acute myocardial infarction (AMI), cardiac arrest, ventricular fibrillation, and stroke. Secondary outcomes included cardiac complications, hemorrhagic complications, pulmonary complications, hospitalization costs, and length of stay (LOS). Detailed definitions of outcomes are provided in Table 2.

**Table 2:** Short-term outcomes following regular vs hybrid ablation for atrial fibrillation

Complication	Before PSW			After PSW		
	Regular ablation	Hybrid ablation	*P*-value	Regular ablation	Hybrid ablation	*P*-value
	N = 47,015	N = 1925		N = 48,007	N = 50,889	
MACE	2640 (5.62%)	85 (4.42%)	.2414	2688 (5.6%)	1578 (3.1%)	.0036
Stroke/TIA	1575 (3.35%)	65 (3.38%)	.9785	1584 (3.3%)	1298 (2.5%)	.33
Cardiac complications	3490 (7.4%)	155 (8.1%)	.721	3601 (7.5%)	4488 (8.8%)	.594
Acute MI	680 (0.014%)	NR	----	687 (1.4%)	122 (0.2%)	.0048
Pericardial effusion	1905 (4.1%)	NR	---	1963 (4.1%)	509 (1.0%)	.0061
Tamponade	785 (1.7%)	0 (0%)	<.001	792 (1.7%)	0 (0.0%)	<.001
Pericardiocentesis/drain	1165 (2.5%)	90 (4.7%)	.0496	1200 (2.5%)	3170 (6.2%)	.0293
Pulmonary complications	710 (1.5%)	65 (3.4%)	.0041	725 (1.5%)	1919 (3.8%)	.0987
Hemorrhagic complications	1885 (4.0%)	285 (14.8%)	<.001	1901 (4.0%)	6437 (12.7%)	<.001
Operative hemorrhage	1455 (3.1%)	250 (13.0%)	<.001	1479 (3.1%)	4748 (9.3%)	<.001
Blood transfusion	820 (1.7%)	65 (3.4%)	.1384	811 (1.7%)	2,163 (4.2%)	.1528
Infectious complications	1765 (3.8%)	NR		1786 (3.7%)	1389 (2.7%)	.5945
Length of stay (mean ± SD)	3.19 ± 2.20	3.53 ± 1.61	.023	3.19 ± 2.45	2.96 ± 0.30	.285
Total charges (mean ± SD)	$160,014 ± 68,786	$203,886 ± 80,956	<.001	$160,588 ± 77,025	$185,480 ± 15,226	.0041

MACE = major adverse cardiovascular event; MI = myocardial infarction; NR = non-reportable; PSW = propensity score weighting; SD = standard deviation; TIA = transient ischemic attack.

### Statistical analysis

STATA, version 17 (Stata Corp, College Station, TX), was used to conduct the analysis. Trend weight files provided by the Agency of Healthcare Research and Quality were used to reflect national estimates. The χ^2^ test and Wilcoxon rank-sum test were used to compare categorical variables and continuous variables, respectively. We ran a logistic regression model to evaluate the probability of each patient belonging to different groups, adjusting for key demographic, clinical, and hospital-related variables. These included age, sex, and major comorbidities such as AF type, hypertension, diabetes, congestive heart failure, peripheral vascular disease, renal failure, liver disease, chronic obstructive pulmonary disease, hypothyroidism, lymphoma, obesity, alcohol abuse, and paralysis. Additionally, we accounted for socioeconomic and hospital factors, including urban-rural classification, race, hospital bed size, teaching status, and geographic region. To enhance the reliability of our model in the face of variable data, we integrated robust standard errors. To achieve a balanced comparison between the 2 groups, we calculated and applied inverse propensity scores. This technique allowed us to assign specific weights to participants: those in the intervention group received weights based on the inverse of their propensity scores, while control-group members were weighted inversely to the likelihood of not receiving the intervention.^11^ After that, we calculated the standardized mean differences (SMDs) for key variables, finding that all SMD values were less than 0.1, indicating well-balanced groups. This innovative weighting approach helped us effectively control for confounding factors, enhancing the credibility of our findings. Propensity score weighting (PSW) has an advantage over propensity score matching in retaining the entire sample and thus maintaining statistical power.^12^^,^^13^ The resulting weights were used to adjust for potential confounding in subsequent analyses. A univariate logistic regression analysis using all variables and comorbidities in Table 1 was used to calculate unadjusted odds ratios (ORs) for the primary outcomes. Subsequently, a multivariate logistic regression analysis was conducted, including variables that were both clinically relevant and statistically significant (*P <* .1) in the univariate analysis, to compute adjusted ORs for the primary outcomes, thereby accounting for confounding factors. We reported this study according to the Strengthening the Reporting of Observational Studies in Epidemiology guidelines for observational studies.^14^

## Results

**Figure 2:**
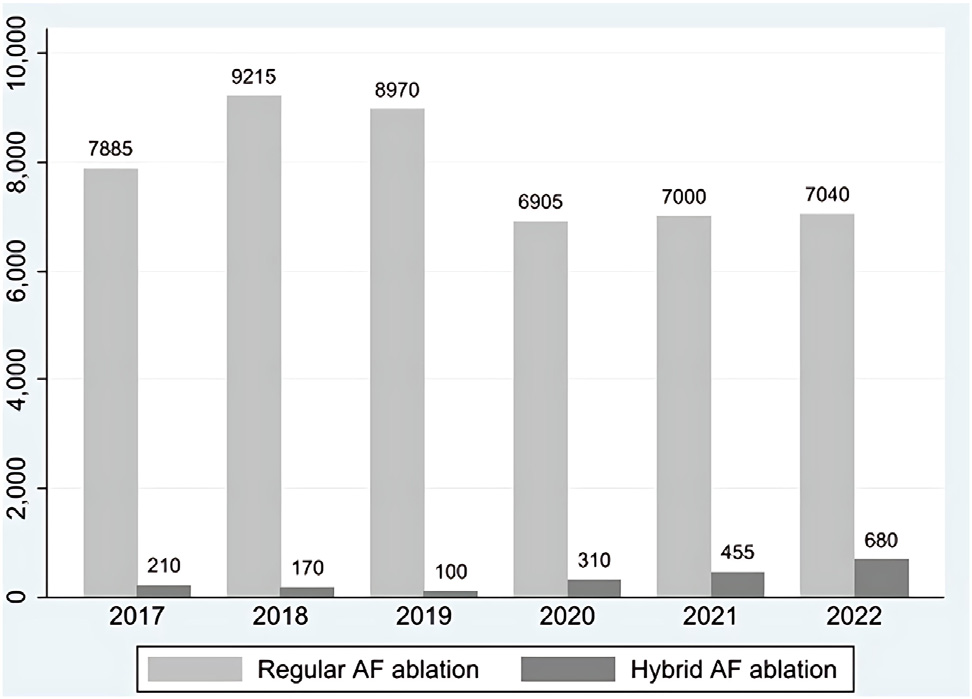
Trends in regular vs hybrid atrial fibrillation (AF) ablation procedures from 2017 to 2022.

**Figure 3:**
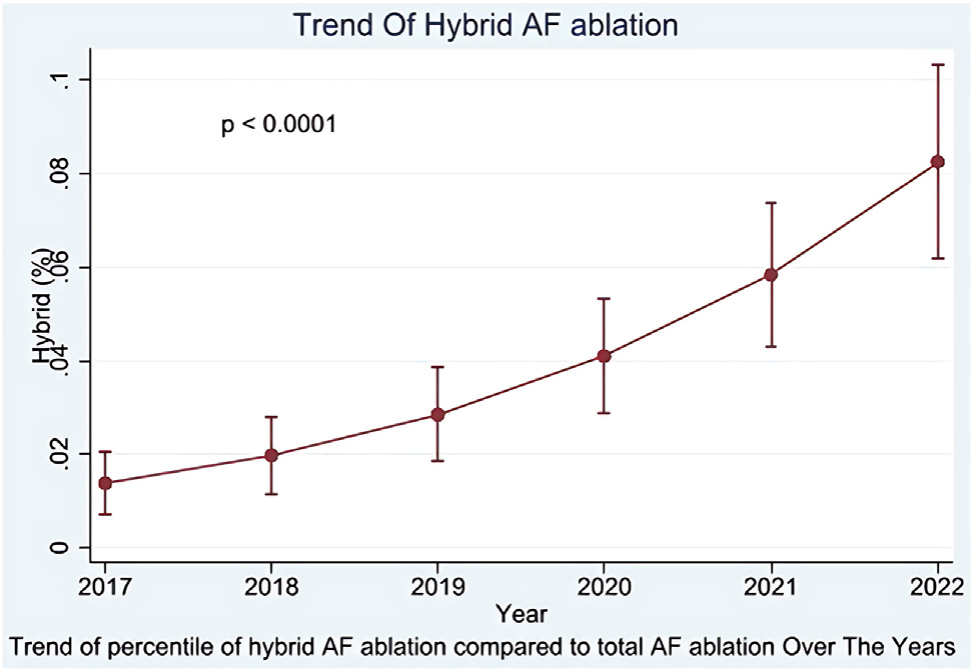
Trend in the percentage of hybrid AF ablation compared with total AF ablation (2017–2022). AF = atrial fibrillation.

Between 2017 and 2022, the use of hybrid ablation showed a notable upward trend, rising from 2% of total AF ablation in 2017 to 8% in 2022 (Figures 2 and 3).

### Baseline characteristics

From January 2017 to December 2022, a total of 48,940 weighted AF ablation cases were identified in the US, with 1925 patients (3.9%) undergoing hybrid AF ablation and 47,015 patients (96.1%) undergoing ECA. Patients undergoing hybrid procedures were significantly younger (mean age: 65.7 vs 67.8 years, *P <* .001) and less likely to be women (29% vs 43% *P <* .001). They were also more likely to be obese (41.8% vs 27.6%, *P <* .001), and their admissions were predominantly elective (92.2% vs 57.8%, *P <* .001). In contrast, ECA ablation patients had a higher prevalence of peripheral vascular disease (13.6% vs 9.6%, *P =* .002), chronic obstructive pulmonary disease (21.5% vs 16.4%, *P =* 0.004), and liver disease (2.5% vs 1%, *P =* .003). Detailed baseline characteristics are provided in Table 1. After PSW, there were no statistical differences between the 2 groups, as all SMD values were less than 0.1, indicating well-balanced groups.

### Clinical outcomes

**Figure 4:**
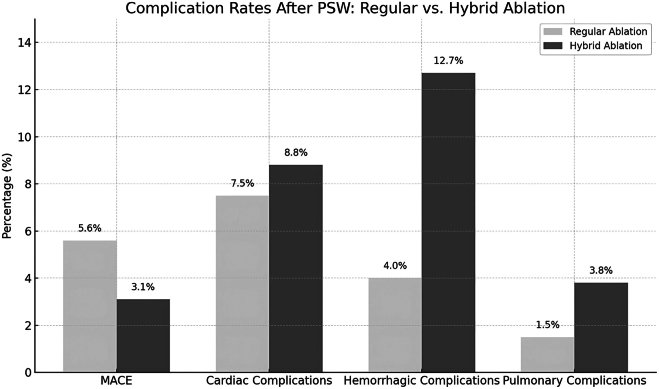
Complication rates after PSW: regular vs hybrid ablation. This bar chart compares the rates of major complications between regular endocardial catheter ablation and hybrid ablation after propensity score weighting. The hybrid group showed a significantly lower rate of MACE but higher rates of hemorrhagic and pulmonary complications. Cardiac complication rates were similar between groups. Values are shown as percentages. MACE = major adverse cardiovascular event; PSW = propensity score weighting.

After PSW, the MACE rate was significantly lower in the hybrid ablation group compared with the endocardial group (3.1% vs 5.6%, *P =* .0036). The rates of cardiac complications (8.8% vs 7.5%, *P =* .594), stroke (2.6% vs 3.3%, *P =* .330), and infections (2.7% vs 3.7%, *P =* .595) were comparable between groups. However, AMI was significantly less frequent in the hybrid group (0.2% vs 1.4%, *P =* .0048). Pericardial complications showed notable differences: the hybrid group experienced lower rates of pericardial effusion (1.0% vs 4.1%, *P =* .0061) and tamponade (0% vs 1.65%, *P <* .001), but had a higher incidence of peri-cardiocentesis or peri-cardial drain placement (6.2% vs 2.5%, *P =* .0293) (Table 2) (Figure 4).

Pulmonary complications were more frequent in the hybrid group (3.8% vs 1.5%, *P =* .099), though this difference did not reach statistical significance. Hemorrhagic complications were significantly higher with hybrid ablation (12.7% vs 4.0%, *P <* .001), including operative hemorrhage (9.3% vs 3.1%, *P <* .001). Despite these differences, blood transfusion rates were similar between groups (4.2% vs 1.7%, *P =* .1528). The mean LOS was comparable (2.96 ± 0.30 vs 3.19 ± 2.45 days, *P =* .285), while total hospital charges were significantly higher in the hybrid group ($185,480 ± 15,226 vs $160,588 ± 77,025, *P =* .0041) (Table 2) (Figure 4).

**Figure 5:**
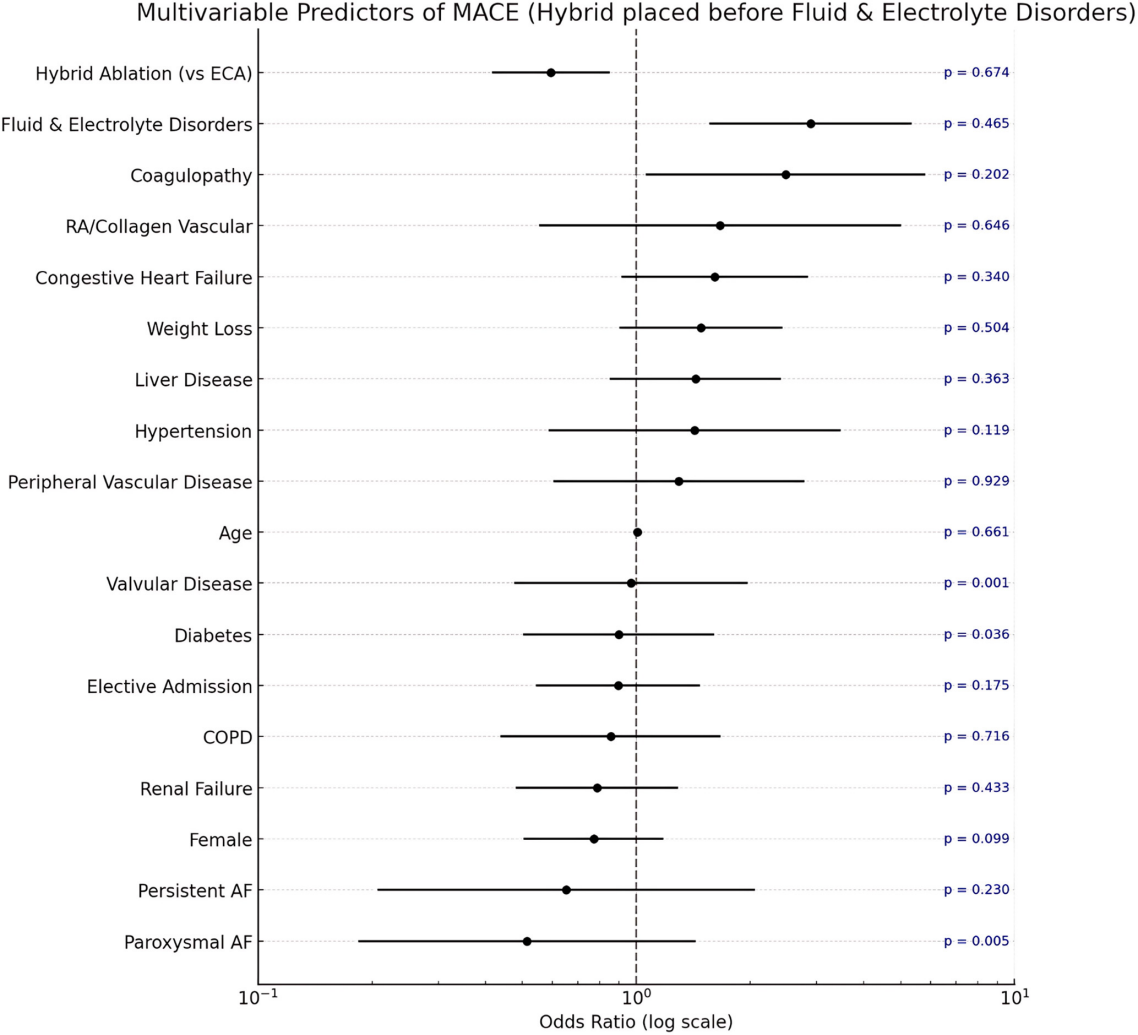
Multivariable analysis of major adverse cardiac events (MACEs) after propensity score weighting (PSW). AF = atrial fibrillation; COPD = chronic obstructive pulmonary disease; ECA = endocardial catheter ablation.

In the adjusted multivariable analysis for MACE, hybrid ablation was associated with a significantly lower risk compared with ECA (OR 0.59, 95% confidence interval [CI]: 0.42–0.85, *P =* .005). Coagulopathy was independently associated with increased risk (OR 2.48, 95% CI: 1.06–5.80, *P =* .036), as were fluid and electrolyte disorders (OR 2.89, 95% CI: 1.56–5.35, *P =* .001), which encompass abnormalities in sodium, potassium, acid–base balance, and volume status. Age, sex, heart failure, and AF subtype were not significant predictors in this model (Figure 5).

Over the course of the study period, we observed an increase in the rate of hybrid AF ablation in comparison to ECA (Figures 2 and 3). The proportion of hybrid ablation cases rose significantly, starting at 2.6% in 2017 and reaching nearly 8.8% by 2022. Similarly, the total number of patients undergoing hybrid ablation grew from 210 in 2017 to 680 in 2022 (Figure 2).

## Discussion

Hybrid ablation is a growing technique for AF ablation that combines both endocardial and epicardial ablation approaches. This study is the largest and first analysis to leverage the NIS database to assess the in-hospital safety and trends of hybrid AF ablation compared with ECA.

Our study revealed the following:

1. Between 2017 and 2022, the use of hybrid ablation increased from 2.6% to 8.8% of all AF ablation procedures (Figures 2 and 3).
2. Before PSW, we found a comparable rate of MACE between the 2 groups but after PSW we found the rate of MACE is significantly lower in the hybrid AF ablation group.
3. The rates of cardiac complications, stroke, infections, and blood transfusion were similar between both groups.
4. AMI, pericardial effusion, and cardiac tamponade were all less frequent in the hybrid group.
5. Pulmonary and hemorrhagic complications were more common in the hybrid group.
6. The mean LOS was comparable between the 2 groups.

Our pre-PSW findings are consistent with more recent studies showing comparable safety between hybrid ablation and ECA. The CEASE-AF trial, a multicenter randomized study comparing staged hybrid ablation versus catheter ablation in persistent AF, found no significant difference in major complications between groups (7.8% vs 5.8%, *P* = .75). Similarly, the HARTCAP-AF trial, conducted across 14 European centers in long-standing persistent AF, reported comparable rates of serious adverse events (21% vs 14%, *P* = .685).^15^^,^^16^ Notably, these studies employed bipolar epicardial ablation. In contrast, earlier studies using unipolar energy during the epicardial phase reported higher complication rates. Edgerton and colleagues^17^ (2015, n = 59) observed significantly higher 12-month complication and mortality rates in the hybrid group (20.8% vs 2.9%, *P =* .036), and the CONVERGE trial similarly showed a higher 30-day major adverse event rate with hybrid ablation (7.8% vs 0%, *P =* .052).^18^

A possible explanation for the higher complication rates observed in the hybrid arm of earlier studies is the use of unipolar radiofrequency energy during the epicardial ablation phase. This technique has been associated with less controlled lesion formation and a greater risk of pericardial complications. In contrast, more recent studies utilizing bipolar ablation, such as CEASE-AF and HARTCAP-AF, have reported complication rates for hybrid ablation that are comparable with endocardial-only approaches, suggesting that advances in energy delivery may have improved the safety profile of the hybrid strategy.^19^, ^20^, ^21^ Pulsed field ablation has emerged as a safer, non-thermal alternative to traditional AF ablation. However, it was not in routine use during our study period (2016–2022). As such, our findings offer a relevant pre- pulsed field ablation benchmark, particularly in higher-risk, inpatient populations.^22^ Additionally, operator experience has likely improved over time, contributing to better outcomes.

After PSW, the hybrid ablation group demonstrated a significantly lower rate of MACE compared with the endocardial group. This shift likely reflects the effect of adjusting for baseline differences, as the hybrid cohort initially had a higher comorbidity burden. These findings suggest that the higher unadjusted MACE rates in the hybrid group were more reflective of underlying clinical complexity rather than procedural risk. Moreover, approximately 13% of the hybrid group had paroxysmal AF, a population typically excluded from prior hybrid ablation trials.^15^^,^^16^ Because paroxysmal AF is associated with lower procedural complexity, its inclusion may have influenced outcomes—particularly as the difference in MACE was not significant before adjusting for AF type.^23^ Additionally, because the NIS database only captures inpatient encounters, many lower-risk patients undergoing same-day endocardial ablation in the outpatient setting are not represented. As a result, the inpatient endocardial cohort may reflect a sicker population, which could contribute to the observed differences in outcomes.

The robustness of our findings was further confirmed by the adjusted multivariable analysis, which supported our earlier observations of lower complication rates with hybrid ablation. It also emphasized the importance of patient selection and preprocedural optimization, as coagulopathy and fluid and electrolyte disturbances remained independently associated with an increased risk of MACE.

In the HARTCAP-AF trial, which included 41 patients, none experienced an AMI.^16^ Notably, other studies have not specifically addressed the incidence of AMI in the context of hybrid AF ablation.^20^^,^^24^ In our analysis, we observed a significantly lower incidence of AMI in the hybrid ablation group compared with the ECA group (0.37% vs 1.44%, *P <* .001). This finding may initially be attributed to patient selection, as the hybrid group included younger patients with fewer comorbidities and a higher proportion of elective admissions. Importantly, this difference persisted even after PSW and achieving an SMD of less than 1% between the 2 groups, suggesting other contributing factors. The method of energy delivery during hybrid ablation may play a role. The bipolar radiofrequency technique commonly used in hybrid procedures offers more controlled and precise myocardial injury compared with the unipolar technique, potentially minimizing collateral damage, and associated ischemic complications.^19^, ^20^, ^21^ Stroke rates were similar between the 2 groups (3.32% vs 3.35%, *P =* .971), consistent with findings from van der Heijden and colleagues^21^ meta-analysis (1.7% vs 1.1%, *P =* .302). The higher incidence observed in our study is likely because of reporting bias inherent to the NIS database.

Our study found that the hybrid group had a higher rate of pericardial drain placement compared with the ECA group. This likely reflects routine drain use during the Convergent hybrid procedure rather than a true complication. In these procedures, pericardial drains are typically placed prophylactically as part of surgical access to the epicardium.^25^ Importantly, this routine drain placement may reduce the risk of significant pericardial effusion or tamponade—findings that align with our observation of lower rates of both complications in the hybrid group. The more clinically relevant signals may lie in the rates of pericardiocentesis and transfusion, which could reflect unanticipated pericardial bleeding, potentially related to intra-procedural anticoagulation or surgical access.

Our study found a significantly higher rate of hemorrhage in the hybrid ablation group compared with the ECA group (11.71% vs 3.07%, *P <* .001). This finding aligns with previous research. Likewise, a meta-analysis by van der Heijden and colleagues^21^ reported increased bleeding rates in hybrid ablation compared with ECA (1.6% vs 0.4%, *P*  =  .000). The higher rate of hemorrhage can be attributed to the invasive nature of thoracoscopy, which involves tissue dissection and manipulation, thereby increasing the risk of bleeding. After thoracoscopy, patients proceed to the endocardial phase of the procedure, during which heparin is administered.^26^ This anticoagulation, combined with the recent surgical trauma, further amplifies the risk of bleeding. Moreover, the extended duration of hybrid procedures often requires prolonged exposure to anticoagulation, increasing the likelihood of hemorrhagic complications.

Pulmonary complications were trending toward higher frequency in the hybrid ablation group (3.8% vs 1.5%, *P =* .0987), which aligns with a meta-analysis by van der Heijden and colleagues^21^ reported higher rates of pulmonary complications like pneumothorax with hybrid ablation compared with ECA (1.0% vs 0.4%, *P*  =  .033). This trend may be due to the prolonged anesthesia duration required for hybrid procedures, which is a known risk factor for pulmonary complications.^27^ Moreover, the thoracoscopic component involves pleural manipulation, increasing the likelihood of complications such as pneumothorax and pleural effusion. The higher frequency of pericardial drain placement in the hybrid group may also contribute to reduced respiratory effort post-procedure, further elevating the risk of pulmonary complications.^27^

The use of hybrid ablation has steadily increased, from 2.6% of AF ablations in 2017 to 8.8% in 2022. This trend reflects growing familiarity with the procedure, advancements in technology, and its expanding role in managing complex AF cases. The increase in the hybrid ablation ratio may also be influenced by a decline in endocardial ablations, particularly during and after the coronavirus disease 2019 pandemic.^28^ Hybrid ablation’s ability to address limitations of conventional methods, such as incomplete lesion formation, likely contributes to its rising popularity. The LOS was comparable between the ECA and hybrid groups (3.19 ± 2.45 vs 2.96 ± 0.30 days, *P =* .285). Both were shorter than what was reported in a prior 2019 NIS study, which found an average LOS of approximately 4.7 days.^29^ This likely reflects improvements in procedural efficiency and evolving patient selection in more recent practice. However, it is important to note that the NIS only captures inpatient procedures. As outpatient AF ablations are increasingly common, particularly in lower-risk patients, our cohort may be skewed toward sicker individuals, potentially affecting LOS estimates—especially in the endocardial group.

## Limitations

This study has several limitations. First, the retrospective nature of the analysis and reliance on administrative coding makes it susceptible to errors. Second, the NIS database does not include important clinical details, such as hemodynamic parameters, medication use, imaging findings, or laboratory results. Information about the duration of AF, baseline echocardiographic findings, or procedural specifics, including surgical techniques, ablation techniques, or staging, was also not available. The NIS does not capture outpatient procedures or link multiple admissions, so we could not include staged hybrid ablations or left atrial appendage closures done outside the index hospitalization. We could not distinguish between different hybrid techniques because of ICD-10 coding limits, so our findings likely reflect convergent procedures but may not apply to all hybrid strategies. Outpatient AF ablations are not captured in the NIS, which may bias our endocardial group toward higher-risk, hospitalized patients. Finally, efficacy outcomes, such as long-term freedom from AF, were not included in this analysis. Despite these limitations, our findings provide valuable insights into the comparative safety and resource utilization of hybrid AF ablation.

Despite its limitations, the use of a large national database ensures broad generalizability of these findings across diverse patient populations.

## Conclusion

Hybrid AF ablation demonstrated a lower MACE rate compared with ECA, reflecting improved safety outcomes over earlier reports. This may be partly attributed to advancements in technique. Although overall cardiac complications and transfusion rates were similar, hybrid ablation was associated with higher rates of pulmonary and hemorrhagic complications. These findings highlight the evolving safety profile of hybrid ablation, the need for careful patient selection, and optimized perioperative management.
