# Supplementary material for: In-hospital complications of hybrid vs endocardial atrial fibrillation ablation
Source: Heart Rhythm O2. 2025 May 23;6(9):1259–67. doi: 10.1016/j.hroo.2025.05.023 (PMC12635746; doi:10.1016/j.hroo.2025.05.023)
Supplement: Supplementary Data [file mmc1.docx]

# ICD-10 Codes for Procedures and Conditions

| Category | ICD-10 Codes |
| --- | --- |
| Atrial Fibrillation (AF) | I480, I481, I482, I4891, I4811, I4819, I4820 |
| Epicardial AF Ablation | 02563ZZ, 02573ZZ, 02583ZZ, 025S3ZZ, 025T3ZZ |
| Endoscopic AF Ablation | 02564ZZ, 02574ZZ, 02584ZZ, 025S4ZZ, 025T4ZZ |
| Hybrid AF Ablation | Defined as cases with both Epicardial AF ablation and endoscopic approach |
| Codes for Exclusion |  |
| ICD Placement (Defibrillator) | 02H40KZ, 02H43KZ, 02H44KZ, 02H60KZ, 02H63KZ, 02H64KZ, 02H70KZ, 02H73KZ, 02H74KZ, 02HK0KZ, 02HK3KZ, 02HK4KZ, 02HL0KZ, 02HL3KZ, 02HL4KZ, 02HN0KZ, 02HN3KZ, 02HN4KZ, 0JH608Z, 0JH609Z, 0JH60FZ, 0JH638Z, 0JH639Z, 0JH63FZ, 0JH808Z, 0JH809Z, 0JH838Z, 0JH839Z, 0JWT0FZ, 0JWT3FZ, 0JWTXFZ, 02PA0MZ, 02PA0NZ, 02PA0QZ, 02PA0YZ, 02PA32Z, 02PA3CZ, 02PA3DZ, 02PA3MZ, 02PA3NZ, 02PA3QZ, 02PA3YZ, 02PA42Z, 02PA4MZ, 02PA4NZ, 02PA4QZ, 02PA4YZ, 02PAX2Z, 02PAXMZ, 02PY02Z, 02PY0YZ, 02PY32Z, 02PY3YZ, 02PY42Z, 02PY4YZ, 02PYX2Z, 0JWT02Z, 0JWT0PZ, 0JWT32Z, 0JWT3PZ, 0JPT0PZ, 0JPT3PZ |
| Pacemaker placement | 02H60JZ, 02H60MZ, 02H60NZ, 02H63JZ, 02H63MZ, 02H63NZ, 02H64JZ, 02H64MZ, 02H64NZ, 02HK0JZ, 02HK0MZ, 02HK0NZ, 02HK3JZ, 02HK3MZ, 02HK3NZ, 02HK4JZ, 02HK4MZ, 02HK4NZ, 02580ZZ, 02HN0JZ, 02HN0MZ, 02HN3JZ, 02HN3MZ, 02HN4JZ, 02HN4MZ |
| Supraventricular Tachycardia (SVT) | I471 |
| Nonspecific Tachycardia | I479 |
| Atrial Flutter | I483, I484, I4892 |
| Wolff-Parkinson-White Syndrome (WFW) | I456 |
| Premature Atrial Contractions (PAC) | I491, I492, I4940, I4949 |
|  |  |
| Outcomes |  |
| Myocardial Infarction | I21.x, I22.x, I25.x |
| Cardiac Arrest | I462, I468, I469 |
| Ventricular Fibrillation | I4901, I4902 |
| Stroke/Cerebrovascular Disease | G45.x, G46.x, I60.x-I69.x |
|  |  |
| Cardiac Complications |  |
| Pericardiocentesis or Pericardial Drain | 0W9D00Z, 0W9D0ZX, 0W9D0ZZ, 0W9D30Z, 0W9D3ZX, 0W9D3ZZ, 0W9D40Z, 0W9D4ZX, 0W9D4ZZ |
| Pericardiotomy | 02BN0ZX, 02BN0ZZ, 02BN3ZX, 02BN3ZZ, 02BN4ZX, 02BN4ZZ, 02CN0ZZ, 02CN3ZZ, 02CN4ZZ, 025N0ZZ, 025N3ZZ, 025N4ZZ, 02QN0ZZ, 02QN3ZZ, 02QN4ZZ, 02NN0ZZ, 02NN3ZZ, 02NN4ZZ, 0WCD0ZZ, 0WCD3ZZ, 0WCD4ZZ, 0WCDXZZ |
| Hemopericardium | I312, S2600XA, S2600XD, S2600XS, S2601XA, S2601XD, S2601XS, S26020A, S26020D, S26020S, S26021A, S26021D, S26021S, S26022A, S26022D, S26022S, S2609XA, S2609XD, S2609XS, S2690XA, S2690XD, S2690XS, S2691XA, S2691XD, S2691XS, S2692XA, S2692XD, S2692XS, S2699XA, S2699XD, S2699XS |
| Ventricular Fibrillation and Flutter | I4901, I4902 |
| Ventricular Tachycardia (VT) | I470, I472 |
| Pericarditis | I300, I308, I309 |
| Cardiac Tamponade | I314 |
| Cardiac Effusion | I313 |
| Intra-aortic Balloon Pump | 5A02110, 5A02210 |
| Percutaneous Ventricular Assist Device | 5A0211D, 5A0221D |
| Accidental Puncture or Laceration During Procedure | D7822, J9572, K9172, L7612, M96821, N9972, T81505A, T81505D, T81505S, T81506A, T81506D, T81506S, T81507A, T81507D, T81507S, I9751, I9752 |
| Cardiac Complications from a Procedure | I97710, I97790, I9788, I9789 |
|  |  |
| Pulmonology Complications |  |
| Pneumothorax/hemothorax | J942, J95811, J95812, S270XXA, S270XXD, S270XXS, S271XXA, S271XXD, S271XXS, S272XXA, S272XXD, S272XXS |
| Post-procedural respiratory failure | J95821, J95822, J95830, J95831, J95860, J95861, J95862, J95863, J9588, J9589 |
| Post-procedural aspiration pneumonia | J690, J698, J954, J9589 |
|  |  |
| Hemorrhagic complications |  |
| Post-op hemorrhage requiring transfusion | 30230H0, 30230H1, 30230J0, 30230J1, 30230K0, 30230K1, 30230L0, 30230L1, 30230M0, 30230M1, 30230N0, 30230N1, 30230P0, 30230P1, 30230Q0, 30230Q1, 30230R0, 30230R1, 30230S0, 30230S1, 30230T0, 30230T1, 30233H0, 30233H1, 30233J0, 30233J1, 30233K0, 30233K1, 30233L0, 30233L1, 30233M0, 30233M1, 30233N0, 30233N1, 30233P0, 30233P1, 30233Q0, 30233Q1, 30233R0, 30233R1, 30233S0, 30233S1, 30233T0, 30233T1, 30240H0, 30240H1, 30240J0, 30240J1, 30240K0, 30240K1, 30240L0, 30240L1, 30240M0, 30240M1, 30240N0, 30240N1, 30240P0, 30240P1, 30240Q0, 30240Q1, 30240R0, 30240R1, 30240S0, 30240S1, 30240T0, 30240T1, 30243H0, 30243H1, 30243J0, 30243J1, 30243K0, 30243K1, 30243L0, 30243L1, 30243M0, 30243M1, 30 |
| Post-operative hemorrhage | D62, I97610, I97618, I97620 |
|  |  |
| Infectious complications |  |
| fever | R5081, R5082, R509 |
| Septicemia | B950, B951, B952, B953, B954, B955, B9561, B9562, B957, B958, B960, B961, B9620, B9621, B9622, B9623, B9629, B963, B964, B965, B966, R6520, R6521, T8112XA, T8112XD, T8112XS, T8144XA, T8144XD, T8144XS |

**All cardiac complications** includes Acute MI, cardiac tamponed, pericardiocentesis, pericardiotomy, hemopericardium, cardiac arrest, IABP placement, open heart repair surgery, VF, VT,

**Hemorrhagic complications** includes blood transfusion, and post procedure hemorrhage

**Pulmonary complications** includes post procedure respiratory failure, hydrothorax, pneumothorax and post procedure aspiration

**Infections** includes fever, and septicemia

| Comorbidity | ICD-10-CM Codes |
| --- | --- |
| Congestive Heart Failure | I099, I110, I130, I132, I255, I420, I425, I426, I427, I428, I429, I43, I50, P290 |
| Valvular Disease | A520, I05, I06, I07, I08, I091, I098, I34, I35, I36, I37, I38, I39, Q230, Q231, Q232, Q233, Z952, Z953, Z954 |
| Pulmonary Circulation Disorders | I26, I27, I280, I288, I289 |
| Peripheral Vascular Disease | I70, I71, I731, I738, I739, I771, I790, I792, K551, K558, K559, Z958, Z959 |
| Hypertension | I10, I11, I12, I13, I15 |
| COPD | I278, I279, J40, J41, J42, J43, J44, J45, J46, J47, J60, J61, J62, J63, J64, J65, J66, J67, J684, J701, J703 |
| Rheumatic Disease | M05.x, M06.x, M31.x-M36.x |
| Diabetes | E100, E101, E109, E110, E111, E119, E120, E121, E129, E130, E131, E139, E140, E141, E149, E102, E103, E104, E105, E106, E107, E108, E112, E113, E114, E115, E116, E117, E118, E122, E123, E124, E125, E126, E127, E128, E132, E133, E134, E135, E136, E137, E138, E142, E143, E144, E145, E146, E147, E148 |
| Hypothyroidism | E00, E01, E02, E03, E890 |
| Renal Failure | I120, I131, N18, N19, N250, Z490, Z491, Z492, Z940, Z992 |
| Liver Disease | B18, I85, I864, I982, K70, K711, K713, K714, K715, K717, K72, K73, K74, K760, K762, K763, K764, K765, K766, K767, K768, K769, Z944 |
| Smoking | Z720, F17, Z716 |
| Coagulopathy | D65, D66, D67, D68, D691, D693, D694, D695, D696 |
| Obesity | E66 |
| Weight Loss | E40, E41, E42, E43, E44, E45, E46, R634, R64 |
| Fluid and Electrolyte Disorders | E222, E86, E87 |
| Blood Loss Anemia | D500 |
| Deficiency Anemia | D508, D509, D51, D52, D53 |
| Alcohol Abuse | F10, E52, G621, I426, K292, K700, K703, K709, T51, Z502, Z714, Z721 |
| Dementia | F00.x –F03.x, F05.x, G30.x |
| Paroxysmal Atrial Fibrillation | I48.0 |
| Persistent Atrial Fibrillation | I48.1 |
| Chronic Atrial Fibrillation | I48.2 |
